# Supplementary material for: From sequence to enzyme mechanism using multi-label machine learning
Source: BMC Bioinformatics. 2014 May 19;15:150. doi: 10.1186/1471-2105-15-150 (PMC4229970; doi:10.1186/1471-2105-15-150)
Supplement: Additional file 2 — Java code of ml2db. Additional file ml2db_code.tar.gz contains the Java source code to run the multi-label machine learning experiments and save the results to database. The code’s Javadoc is included. [file 1471-2105-15-150-S2.zip › additional file 2/ml2db/ml2db/doc/mulan/classifier/neural/model/package-use.html]

Uses of Package mulan.classifier.neural.model


JavaScript is disabled on your browser.


- Overview
- Package
- Class
- Use
- Tree
- Deprecated
- Index
- Help

- Prev
- Next

- Frames
- No Frames

- All Classes

# Uses of Package mulan.classifier.neural.model

- Packages that use mulan.classifier.neural.model

  | Package | Description |
  | --- | --- |
  |  |  |
  | --- | --- |
  | mulan.classifier.neural |  |
  | mulan.classifier.neural.model |  |
- Classes in mulan.classifier.neural.model used by mulan.classifier.neural

  Class and Description

  NeuralNet

  Common interface for interaction with a neural network representation.

  Neuron

  Implementation of a neuron unit.
- Classes in mulan.classifier.neural.model used by mulan.classifier.neural.model

  Class and Description

  ActivationFunction

  Abstract base class for activation functions.

  NeuralNet

  Common interface for interaction with a neural network representation.

  Neuron

  Implementation of a neuron unit.

- Overview
- Package
- Class
- Use
- Tree
- Deprecated
- Index
- Help

- Prev
- Next

- Frames
- No Frames

- All Classes
